# Supplementary material for: Integration and evaluation of magnetic stimulation in physiology setups
Source: PLoS One. 2022 Jul 22;17(7):e0271765. doi: 10.1371/journal.pone.0271765 (PMC9307166; doi:10.1371/journal.pone.0271765)
Supplement: S1 File — (ZIP) [file pone.0271765.s001.zip › supplm_repository/magnetometer/hardware/miniMag_sensorshaft_SCH.PDF]

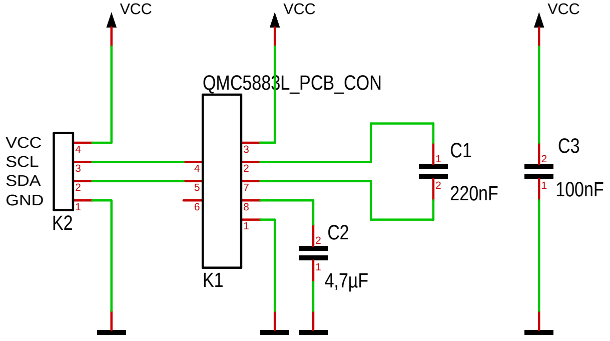

Maßstab	100,00%	UOL	Zeichner M. Ahlers	Blatt 1/1
Änderung	05.01.2022 15:24		Miniature vector magnetometer sensor shaft	
Ausgabe	05.01.2022 15:24			
Datei	miniMag_sensorshaft.T3001		Projekt Miniature vector magnetometer	
