# Supplementary figures and images for: Integration and evaluation of magnetic stimulation in physiology setups
Source: PLoS One. 2022 Jul 22;17(7):e0271765. doi: 10.1371/journal.pone.0271765 (PMC9307166; doi:10.1371/journal.pone.0271765)

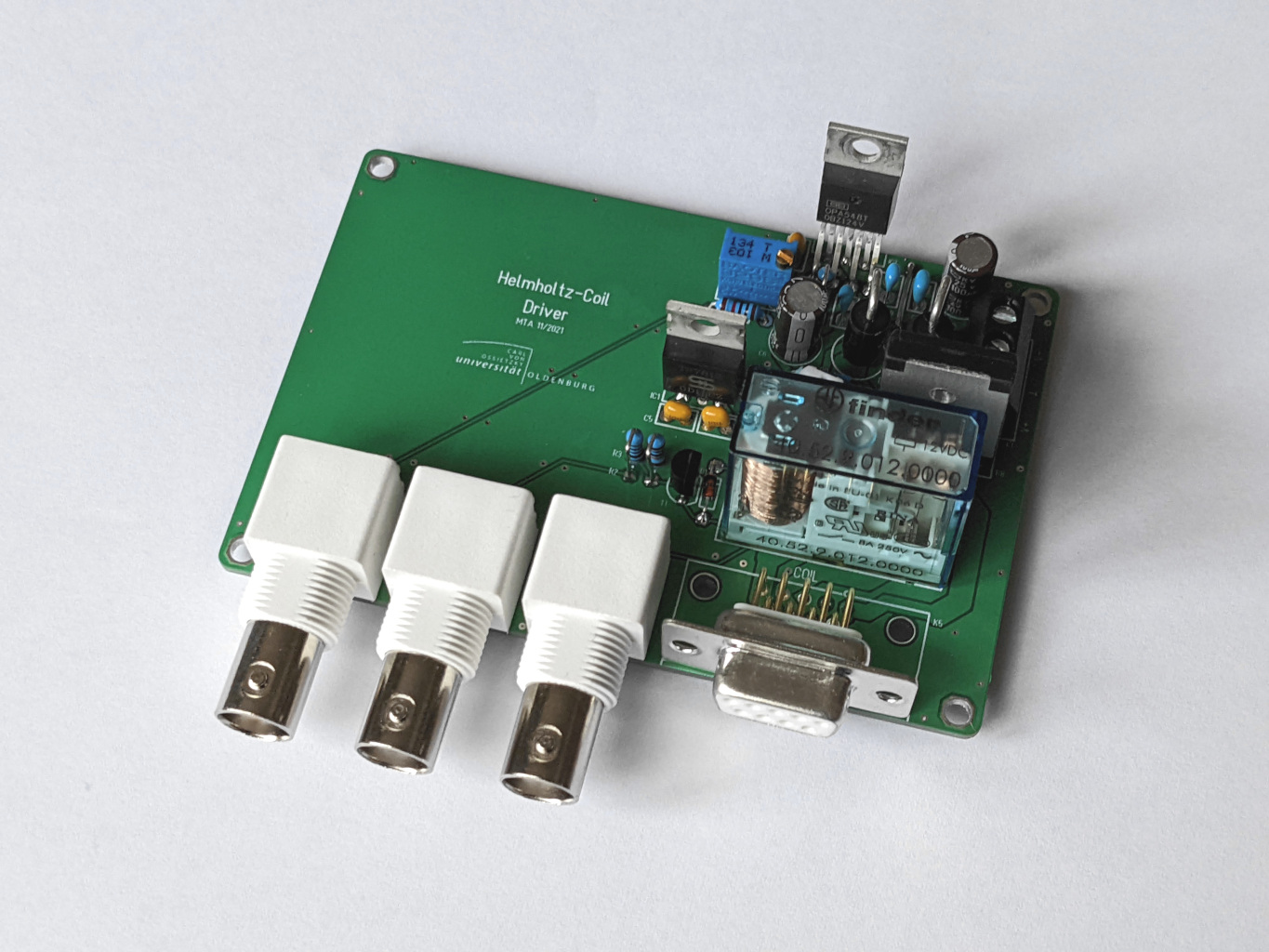

Supplement: S1 File — (ZIP) [file pone.0271765.s001.zip › supplm_repository/img/coilDriver.jpg]

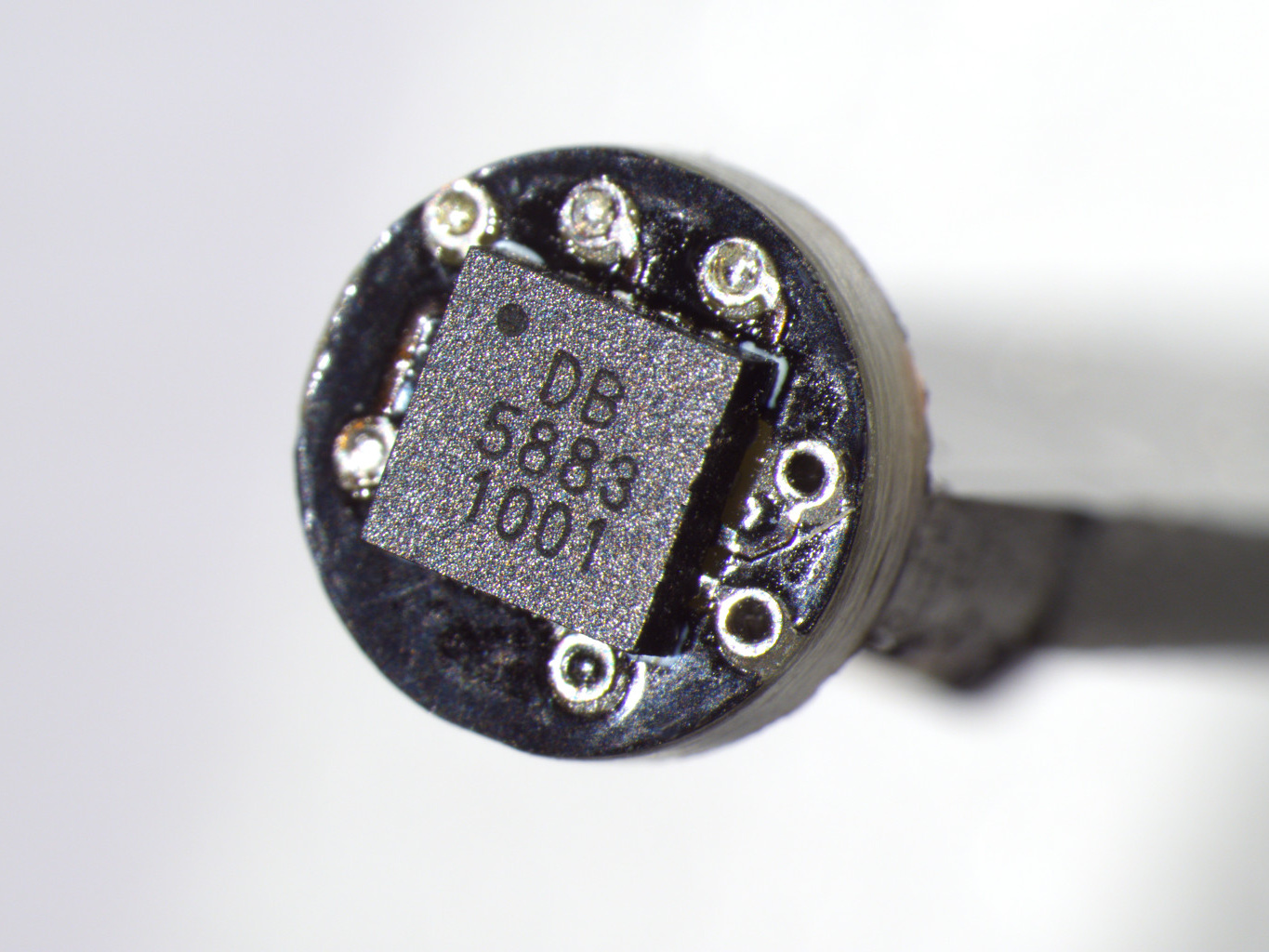

Supplement: S1 File — (ZIP) [file pone.0271765.s001.zip › supplm_repository/img/sensorHead.jpg]

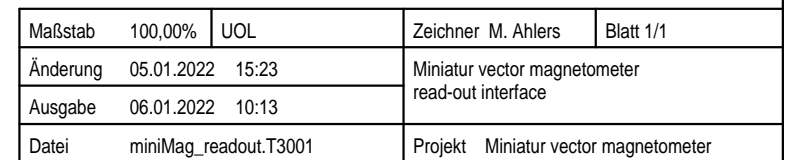

Supplement: S1 File — (ZIP) [file pone.0271765.s001.zip › supplm_repository/magnetometer/hardware/miniMag_readout_SCH.PDF]
